# Integrating *in vitro* metabolomics with a 96-well high-throughput screening platform

**DOI:** 10.1007/s11306-021-01867-3

**Published:** 2022-01-09

**Authors:** Julia M. Malinowska, Taina Palosaari, Jukka Sund, Donatella Carpi, Mounir Bouhifd, Ralf J. M. Weber, Maurice Whelan, Mark R. Viant

**Affiliations:** 1 School of Biosciences, University of Birmingham, Birmingham, B15 2TT UK; 2 European Commission, Joint Research Centre (JRC), 21027 Ispra, Italy; 3 Phenome Centre Birmingham, University of Birmingham, Birmingham, B15 2TT UK; 4 European Chemicals Agency, Helsinki, Finland

**Keywords:** In vitro metabolomics, Direct infusion mass spectrometry, High-throughput screening, Chemical risk assessment, HepaRG, Toxicology

## Abstract

**Introduction:**

High-throughput screening (HTS) is emerging as an approach to support decision-making in chemical safety assessments. In parallel, in vitro metabolomics is a promising approach that can help accelerate the transition from animal models to high-throughput cell-based models in toxicity testing.

**Objective:**

In this study we establish and evaluate a high-throughput metabolomics workflow that is compatible with a 96-well HTS platform employing 50,000 hepatocytes of HepaRG per well.

**Methods:**

Low biomass cell samples were extracted for metabolomics analyses using a newly established semi-automated protocol, and the intracellular metabolites were analysed using a high-resolution spectral-stitching nanoelectrospray direct infusion mass spectrometry (nESI-DIMS) method that was modified for low sample biomass.

**Results:**

The method was assessed with respect to sensitivity and repeatability of the entire workflow from cell culturing and sampling to measurement of the metabolic phenotype, demonstrating sufficient sensitivity (> 3000 features in hepatocyte extracts) and intra- and inter-plate repeatability for polar nESI-DIMS assays (median relative standard deviation < 30%). The assays were employed for a proof-of-principle toxicological study with a model toxicant, cadmium chloride, revealing changes in the metabolome across five sampling times in the 48-h exposure period. To allow the option for lipidomics analyses, the solvent system was extended by establishing separate extraction methods for polar metabolites and lipids.

**Conclusions:**

Experimental, analytical and informatics workflows reported here met pre-defined criteria in terms of sensitivity, repeatability and ability to detect metabolome changes induced by a toxicant and are ready for application in metabolomics-driven toxicity testing to complement HTS assays.

**Supplementary Information:**

The online version contains supplementary material available at 10.1007/s11306-021-01867-3.

## Introduction

A paradigm shift in toxicity testing was triggered by the report *Toxicity Testing in the twenty-first century: A Vision and a Strategy* by the United States National Research Council in 2007 (Krewski et al., 2020; National Research Council, 2007). The report envisioned changes to existing methods recognising that animal-based toxicity testing, usually focused on apical endpoints (e.g. histopathology), holds little relevance to humans and is unfit to test the growing number of chemicals on the market due to its low throughput. In addition, existing approaches were considered expensive, time-consuming and required large numbers of animals. High-throughput screening (HTS) assays were proposed as an alternative due to their ability to rapidly measure the effects of many substances on biological processes in human cells or cell lines using automation (Krewski et al., 2020). Since the release of the report, there have been numerous initiatives contributing towards the utilisation of HTS and its implementation into ecological and human risk assessment (Villeneuve et al., 2019).

In principle, omics technologies align well with the described shift in toxicity testing due to their ability to generate molecular mechanistic information to support decision making in regulatory applications, including chemical grouping and mode of action (MoA) prediction (Sperber et al., 2019; van Ravenzwaay et al., 2016; Viant et al., 2019). The value of metabolomics to risk assessment, defined as the profiling of metabolites (low-molecular-weight biochemicals) in cells, tissues or biofluids, has been acknowledged by several regulatory bodies (European Chemicals Agency, 2016; European Food Safety Authority et al., 2018). When compared to other omics technologies, metabolomics provides measurements of the most downstream molecular changes that are closest to the cellular or organismal phenotype, enabling an association of the measured effects to an adverse outcome (Bouhifd et al., 2013; Taylor et al., 2018). In vitro metabolomics has been demonstrated to predict organ toxicity and identify the MoA of chemicals, which could support regulatory applications and contribute towards the use of non-animal models in toxicity testing (Ramirez et al., 2018). In particular, there has been growing interest in applying in vitro metabolomics to study hepatotoxicity due to the central role of the liver in metabolism of xenobiotics (Cuykx et al., 2018a, 2018b; Mennecozzi et al., 2012; Pomponio et al., 2015; Van den Eede et al., 2015).

In in vitro toxicology studies, reducing the number of cells per sample saves both time and cost, and can increase throughput. However, conventional metabolomics methods such as liquid chromatography–mass spectrometry (LC–MS) typically require more than a million cells per sample (Luo & Li, 2017; Ramirez et al., 2018). Such approaches are not compatible with HT assays using 96-well microplates which utilise volumes of cell suspensions of up to 100 µL, equivalent to approximately only 50,000 cells per well (Thermo Fisher Scientific, n.d.; Villeneuve et al., 2019). Analytical sensitivity could in principle be improved by employing nanoflow LC–MS, however, that significantly decreases sample throughput with chromatographic separations typically requiring 45 min per sample (Chetwynd & David, 2018). Higher throughput may be achieved by applying direct infusion mass spectrometry (DIMS), albeit with the loss of chromatographic separation resulting in more complex mass spectra (Fuhrer et al., 2011; Southam et al., 2017). The development of an automated nanoflow electrospray ionisation (nESI) sample delivery platform for DIMS greatly increased sensitivity, and decreased both ion suppression and sample volumes required (Kempa et al., 2019; Schultz et al., 2000). Sensitivity was further improved (fivefold increase in feature detection) via the spectral stitching nESI method (Southam et al., 2007, 2017). Furthermore, the value of integrating metabolomics with HTS has been demonstrated in drug discovery: Dubuis et al. (2018) employed flow-injection time-of-flight MS to study the effect of exposure of five adherent cell lines (cultured in 96-well microplates) to dichloroacetate, providing insights into its MoA. This approach was also employed to predict the MoA of 212 antimicrobial compounds in *Mycobacterium smegmatis* after creating a library of metabolic responses to 62 reference compounds (Zampieri et al., 2018). Others have focused on more targeted approaches, for example by automating the measurement of adenosine triphosphate in embryonic stem cells to assess the cytotoxicity of compounds (Witt et al., 2021). These developments have provided encouraging results towards the integration of metabolomics with HTS in toxicology.

The objective of this study was to establish and evaluate an in vitro metabolomics–based workflow, employing 50,000 hepatocytes of HepaRG per well, that is fully compatible with a 96-well HTS platform. Sample preparation was semi-automated, with a Biomek FXp automated laboratory workstation used for solvent handling and metabolite extraction. Extracted samples were analysed by nESI-DIMS using a platform comprising a TriVersa NanoMate coupled to an Orbitrap Elite mass spectrometer. To ensure both the high sensitivity and throughput of nESI-DIMS analyses, the metabolomics method described by Southam et al., (2007, 2017) was re-optimised for low biomass samples (50,000 hepatocytes per sample) utilising a Thermo Scientific Orbitrap Elite mass spectrometer. First, the workflow was assessed with respect to sensitivity as well as intra- and inter-plate repeatability of the polar DIMS assays in positive and negative ionisation modes. These assays were then tested by studying the effects of a model toxicant, cadmium chloride, on the metabolome of HepaRG over five exposure times and across three concentrations. To facilitate the application of both metabolomics and lipidomics to this low biomass sample type, the final objective was to implement a lipid extraction method by extending the original solvent system for polar metabolites.

## Methods

### Cell culturing and exposure to CdCl_2_

Culturing of undifferentiated HepaRG cells (HPR101, Biopredic International, Rennes, France, batch HPR-101056) in polystyrene 96-well microplates was conducted as previously described by Joossens et al. (2019) using 5 × 10^4^ cells/well in 100 μL medium. Cell seeding, serial dilution of chemicals and cell treatment were performed in fully automated fashion utilising Hamilton Star and Starlet platforms (Hamilton Italia Srl, Agrate, Brianza, Italy). The liquid handlers were contained in a laminar flow hood and equipped with 96-channel heads. For the study investigating the effect of cadmium chloride (99.99% trace metal basis, Sigma) on the metabolome of HepaRG, three concentrations of the toxicant were used dissolved in dimethyl sulfoxide (DMSO, Sigma) and diluted in cell media (low, medium and high corresponding to 0.42, 1.33 and 4.22 μM, respectively) across five time points (1, 2, 6, 24 and 48 h). The controls in this study were generated by incubating the cells in medium with 0.1% DMSO at each time point. The layouts for each experiment are included in Fig. SI-1 and SI-2. For more information see the Supplementary Information.

### Assessments of sensitivity and intra- and inter-plate metabolic variability

#### Metabolite extraction

Intracellular metabolites were extracted using a monophasic 1:3:1 (v/v/v) water:methanol:chloroform solvent system on a Biomek FXp automated workstation (Beckman Coulter, Indianapolis, IN, USA). Automated labware positioners of the workstation, for handling samples and extraction solvents, were precooled to − 15 °C. Polypropylene 96-well microplates (Thermo Scientific, Loughborough, UK) were used as solvent reservoirs and filled with either methanol (LC–MS grade, Honeywell, Seelze, Germany), water (LC–MS grade, Merck, Darmstadt, Germany) or chloroform (HPLC grade, Merck). Each polystyrene microplate well was extracted with methanol (45 μL) and water (15 μL), including mixing by pipetting 30 μL up/down five times. The extract (40 μL) was transferred to a clean polypropylene 96-well microplate (“collection microplate”), and the original well was re-extracted (30 μL methanol and 10 μL water); this extract (40 μL) was then transferred to the same well of the polypropylene 96-well microplate. Next, chloroform (20 μL) was added to each well of the polypropylene microplate, which was shaken (200 rpm, 120 s, room temperature; BioShake) and centrifuged (3,622*g*, 3 min, 4 °C; Sigma 6-16KL). Finally, supernatants (60 μL) were transferred to a clean polypropylene microplate and dried using a SpeedVac concentrator at 35 °C (SPD111V230, Thermo Scientific Savant). Microplates were stored at − 20 °C until mass spectrometry analyses.

#### Acquisition of DIMS metabolomics data

Individual samples were resuspended for positive ionisation mode (20 μL 4:1 (v/v) methanol:water containing 0.25% (v/v) formic acid (~ 98%, Honeywell)) or negative ionisation mode analyses (20 μL 4:1 (v/v) methanol:25 mM aqueous ammonium acetate (≥ 99.9% trace metal basis, Honeywell)). Intrastudy quality control (QC) samples were generated after sample resuspension by pooling equal volume aliquots of representative wells into a common pool. Metabolomics data were collected using an Orbitrap Elite mass spectrometer (Thermo Scientific) coupled to a chip-based nESI ionisation platform (TriVersa NanoMate, Advion, Ithaca, USA). The DIMS method reported by Southam et al., (2007, 2017) was modified to account for small sample biomass, specifically each sample was analysed as a single infusion using spectral stitching nESI-DIMS with overlapping *m/z* windows each collected four times, here termed internal scan replication (an approach first used for dried blood spot analysis with Liquid Extraction Surface Analysis (Palmer, 2019)). This approach enabled the resuspension volume per sample to be reduced from typically 40 μL to only 20 μL for polar analysis, concentrating the intracellular metabolites extracted from 50,000 hepatocytes. Other DIMS acquisition parameters were as reported previously (Southam et al., 2017). The order of sample analysis was determined by applying block randomisation.

#### Processing and analysis of DIMS metabolomics data

Samples for which electrospray ionisation failed were excluded, based on visual inspection of nESI current and ion injection times. DIMS data were processed and analysed using the DIMSpy tools within the Galaxy workflow management system (Southam et al., 2017; Weber & Zhou, 2020) and R package structToolbox (Lloyd et al., 2020) with standard parameters (see Supplementary Information), except for the variations noted here. This included extensive filtering to remove noise, features that occurred in the extraction blank, and other irreproducible signals. In particular, a feature (or peak) had to be present in at least 3 of the 4 internal scan replicates per sample in order to be retained in the data matrix, replacing the ‘replicate filter’ step previously reported. After processing, probabilistic quotient normalisation, PQN (Dieterle et al., 2006), was applied and the sensitivity and repeatability of the data were evaluated in terms of (1) feature count (for features present in ≥ 80% of samples), (2) count of features with intensity relative standard deviation (RSD) ≤ 30% across specific sample classes, and (3) median RSD (mRSD) across specific sample classes (including the intrastudy QC samples only, providing a measure of analytical repeatability (Parsons et al., 2009)). The assessment of intra-plate variability compared the mRSDs of wells located either at the edge or centre of the microplates (n = 8 per class). Inter-plate variability was evaluated by determining mRSDs of three equivalent microplates (n = 10 per class).

### Pilot study into effects of CdCl_2_ on the HepaRG metabolome

Study samples (described above) were extracted using 1:3:1 (v/v/v) water:methanol:chloroform but with 75% methanol:water pre-made instead of adding them separately, and nESI-DIMS data were acquired and processed as for the assessment of sensitivity and variability study, except that an additional processing step to correct for instrumental drift in signal intensities was applied (see Supplementary Information). To ensure a high-quality dataset, features with RSD values exceeding 30% in the QC samples were discarded as unreliable. In addition, principal component analysis (PCA) (following PQN, imputing missing values using k-nearest neighbour algorithm (k = 5), generalised log transformation and mean centring) was used to identify five outlying samples that were removed, and the data processing was rerun without these outliers. Statistical analyses were conducted using non-imputed data with the R package, structToolbox (Lloyd et al., 2020), including analysis of variance (ANOVA) (with false discovery rate correction) and post-hoc testing (with a correction for unbalanced design) to investigate (separately) the effect of (1) time on the HepaRG metabolome and (2) CdCl_2_ at every time point. PCA plots were used to evaluate trends in the data, including any effects of time and/or treatment.

### Modification of solvent system to extract polar metabolites and lipids

To ensure compatibility of the HTS approach with lipidomics analysis, the solvent system described above was extended to extract both polar metabolites and lipids from 50,000 hepatocytes. Given the low biomass per sample and need to automate potentially thousands of extractions, a biphasic solvent system was deemed inappropriate. Instead, low volume extraction methods that separately recovered polar metabolites (using 1:3:1 (v/v/v) water:methanol:chloroform and 4:1 (v/v) methanol:water) and lipids (using 1:3:1 (v/v/v) water:methanol:chloroform, 2:1 (v/v) methanol:chloroform and 1:1 (v/v) methanol:chloroform) were implemented. The lipid extracts were analysed by the lipid nESI-DIMS assay in positive ionisation mode (detailed protocols are included in Supplementary Information).

### Metabolite Annotation

Datasets corresponding to (1) pilot study into the effects of CdCl_2_ on the HepaRG metabolome (polar negative assay), (2) modification of solvent system to extract polar metabolites (polar positive assay, extraction solvent: 4:1 (v/v) methanol:water), and (3) modification of solvent system to extract lipids (lipid positive assay, extraction solvent: 2:1 (v/v) methanol:chloroform) were putatively annotated using accurate mass by the Python package BEAMSpy (Birmingham mEtabolite Annotation for Mass Spectrometry, https://github.com/computational-metabolomics/beamspy). These annotations are included in the Supplementary Information. The database used for the annotation of polar metabolites was an in-house HMDB-based list of metabolites prepared by Sostare et al. (2021) following the removal of exogenous compounds to reduce the risk of false positive annotations, whilst the database used for the annotation of lipids was LIPID MAPS. The mass error was set to 5 ppm, and the adducts used were: [M + H]^+^, [M + Na]^+^, [M + NH_4_]^+^ for positive ionisation mode, and [M-H]^−^, [M + Cl]^−^, [M + Hac-H]^−^, [M + Na-2H]^−^, [M + K-2H]^−^ for negative ionisation mode.

## Results and discussion

### Sensitivity and intra-/inter-plate metabolic variability of in vitro metabolomics workflow

Workflows were implemented for the analysis of samples containing 50,000 HepaRG hepatocytes and evaluated with respect to their sensitivity and intra- and inter-plate metabolic variability. Here, sensitivity was defined as the total number of features detected in at least 80% of samples, after blank subtraction; i.e. based on reliable features of biological origin, as often used as a surrogate for sensitivity in metabolomics assays employing relative quantification (Viant et al., 2019). While there is no formal threshold for feature detection, previous reports employing nESI-DIMS metabolomics yielded 2000–4000 features in higher biomass samples, e.g. liver extracts or *Daphnia magna* (Southam et al., 2007; Taylor et al., 2009). Variability of the entire workflow from cell culturing and sampling to measurement of the metabolic phenotype was assessed based on mRSD values of all features across biological control samples, with the target set at < 30%, justified below.

**Fig. 1:**
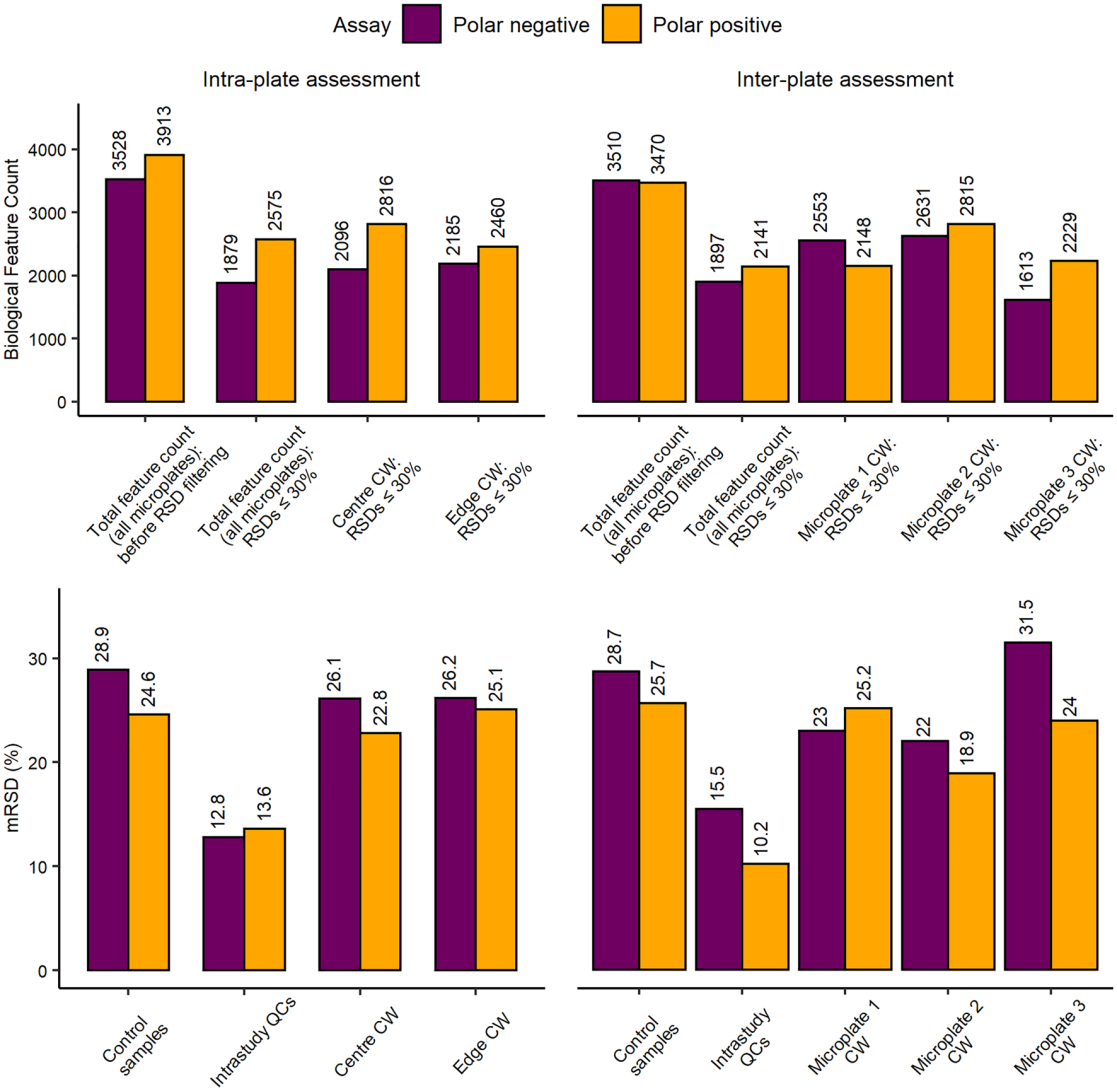
Assessment of relative sensitivity and repeatability of the in vitro HTS nESI-DIMS metabolomics workflow based on a monophasic 1:3:1 (v/v/v) water:methanol:chloroform extraction of 96-well microplates, with 50,000 HepaRG per well within and between 3 microplates for two polar nESI-DIMS assays. CW: control (unexposed) wells of hepatocytes

Here, sensitivity of the polar nESI-DIMS method was deemed sufficient, yielding 3000–4000 features (in ≥ 80% of all samples) in both ionisation modes (Fig. 1). The feature count remained high even after retaining features with intensity RSDs < 30%. This target repeatability was based on previous observations of total (technical and biological) metabolic variability, e.g. mRSDs of 20–22% were reported for larger biomass samples (several million human immortalised K562 cells) using a higher precision detector (nuclear magnetic resonance (NMR) spectroscopy) than employed here (Parsons et al., 2009). The variability of the workflow was further assessed by separately determining the variation of wells located either in the centre or at the edges of a microplate (termed “Intra-plate assessment” on Fig. 1). Both classes met the mRSD threshold of < 30%. Finally, the technical repeatability of data acquisition was highly satisfactory with mRSDs < 15% for the intrastudy QCs in both ion modes (Fig. 1).

Further evaluation of the in vitro metabolomics workflow consisted of a comparison of metabolites extracted from cells cultured in three separate microplates (termed “Inter-plate assessment” on Fig. 1). The results further demonstrate sufficiently high sensitivity, with 1897 (polar negative) and 2141 (polar positive) reproducibly detectable metabolic features across all three microplates with RSDs ≤ 30%. In addition, technical repeatability was also satisfactory with intrastudy QCs producing mRSDs ≤ 20%. When considering each individual microplate, microplate 3 produced a lower peak count and higher mRSD (31.5%) than the set threshold in the polar negative assay. This highlights the importance of evaluating the workflow to determine the experimental variation, with the potential to remove a problematic microplate from a large HTS study, if required. Overall, the proposed workflow was found to meet criteria for sensitivity as well as intra- and inter-plate metabolic variability suggesting that the polar metabolomics assays may be used for complementing HTS platforms.

### Pilot study into effects of CdCl_2_ on the HepaRG metabolome

To evaluate the new HTS metabolomics workflow in a toxicological context, from cell culture to nESI-DIMS metabolomics analysis, a study was undertaken to characterise the baseline metabolism of unexposed HepaRG (i.e. control hepatocytes in cell medium with 0.1% DMSO (v/v)) over time, assessing the sensitivity, repeatability and metabolic perturbations induced by a media change. Secondly, the study sought to detect the metabolic perturbations induced in hepatocytes of HepaRG by a model toxicant, CdCl_2_. Data were collected using the polar assay in positive and negative ionisation modes. The final dataset obtained using the positive ionisation mode resulted in 3481 features and mRSD of 14.7% for the QC samples. The analyses presented below focus on the polar negative nESI-DIMS assay; the final dataset used for statistical analysis demonstrated a higher technical quality than positive ionisation mode, with a feature count of 4983 and mRSD of intrastudy QC samples of only 9.1%.

**Fig. 2:**
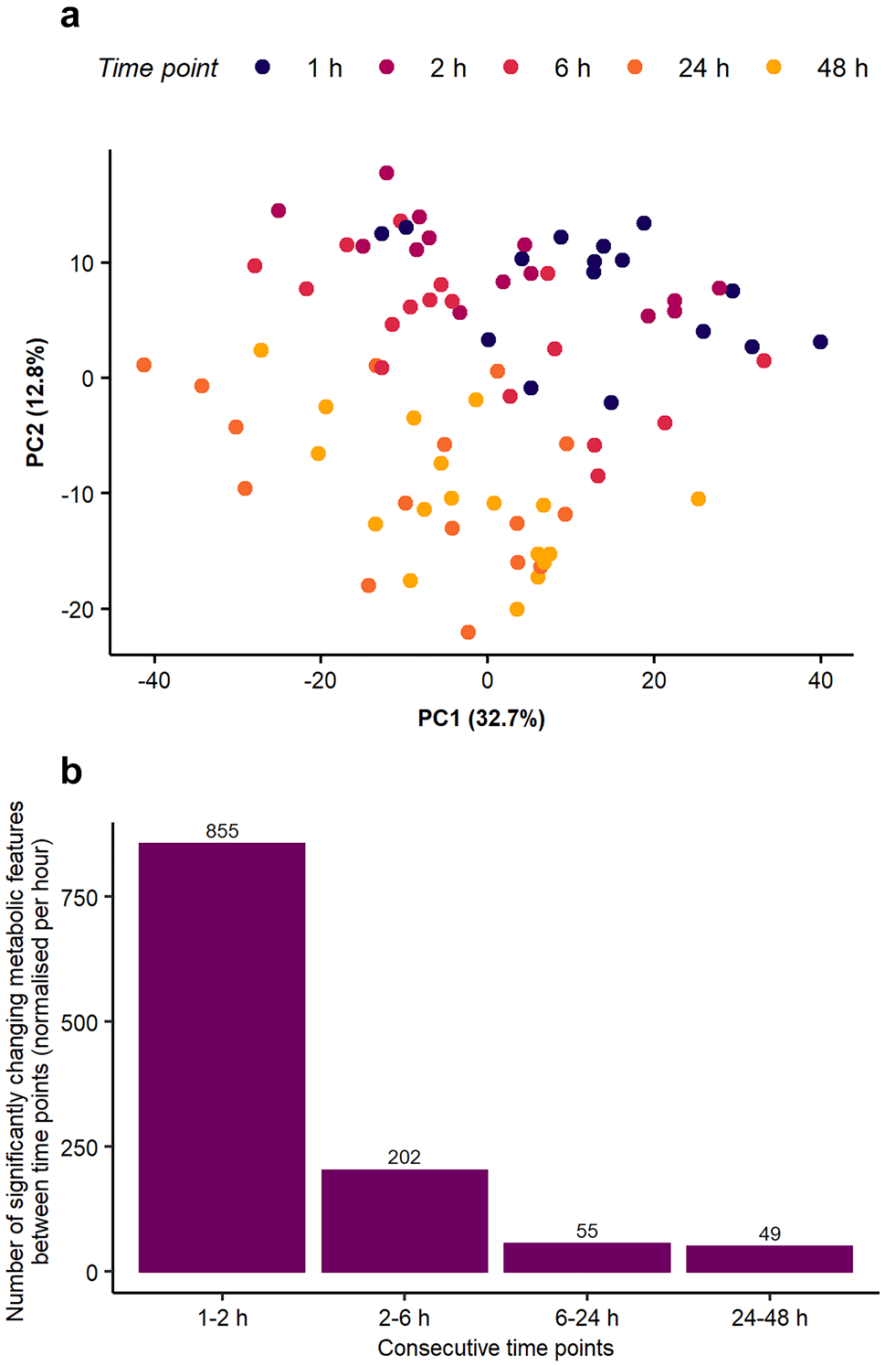
Temporal changes in the metabolome of unexposed HepaRG up to 48 h after a media change: **a** PCA score plot demonstrates changes in the baseline metabolism of the hepatocytes after 1, 2, 6, 24 and 48 h; **b** Number of significantly changing metabolic features between each pair of consecutive time points, normalised to show the number of changes per hour (one-way ANOVA, FDR-corrected p ≤ 0.05 with the Tukey–Kramer method for post-hoc testing as used for unbalanced designs)

The baseline characterisation of metabolism of unexposed cells revealed a temporal shift through the 2-day study, with the PCA score plot (Fig. 2a) showing separation of earlier and later time points (1 and 2 h vs. 24 and 48 h) across the PC2 axis. Additional principal components and their permutations were plotted and included in Fig. SI-3 further supporting separation of earlier and later time points. The dataset was further analysed to determine the number of significantly changing features between each pair of consecutive time points (Fig. SI-4). This representation of the results, however, does not account for the varying time differences between consecutive samplings (i.e. only 1 h spacing between the first two time points, but 24 h between the last two), hence the average number of significantly changing features *per hour* was determined (Fig. 2b). The media change induced large shifts in metabolism in the first few hours, but sometime after ca. 6 h the basal metabolism of HepaRG stabilised. The implications of this observation are significant for HTS studies, specifically that the ability to detect metabolic changes due to toxicant exposure may be greatest after ca. 6 h of exposure, when the baseline metabolome of HepaRG is more consistent. Further analysis of the unexposed cell data revealed that 22 metabolic features changed significantly at all five time points (one-way ANOVA followed by post-hoc analysis, Fig. SI-5). This included putatively annotated endogenous compound, glutathione ([M + K-2H]^−^ adduct, HMDB0000125), which is known to be involved in detoxification of reactive oxygen species, highlighting the importance of time-matched control samples in HTS studies. As shown in Fig. SI-6, even in the biological control data, the intensity of glutathione increases over time (up to 24 h), as did the repeatability of its measurement, with a final decrease in both peak intensity and repeatability at 48 h.

**Fig. 3:**
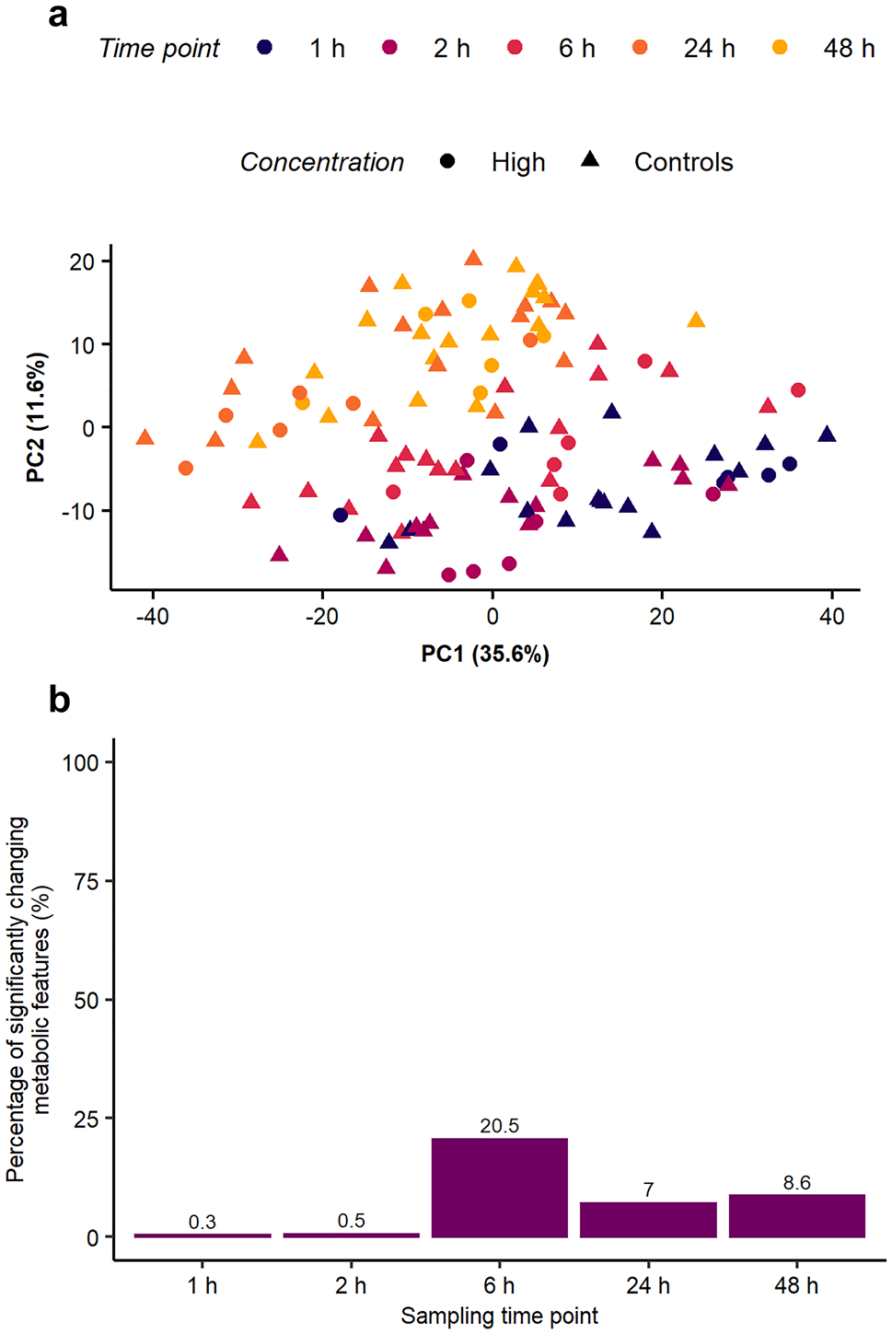
Effect of CdCl_2_ on the metabolome of HepaRG over a 1–48 h exposure period: **a** PCA score plot demonstrates that sampling time (after the media change at time 0 h) exerts a larger effect on unexposed HepaRG metabolism than exposure to the highest concentration of CdCl_2_, **b** Number of metabolic features significantly perturbed by CdCl_2_ at each time point after conducting one-way ANOVA, FDR-corrected p ≤ 0.05 (the values shown include interactions between control and exposed samples (low, medium, high) as well as interactions between exposure groups)

To assess whether the HTS metabolomics workflow had sufficient sensitivity to detect toxicant-induced changes in the metabolome of 50,000 HepaRG hepatocytes, the cell line was exposed to CdCl_2_ at three concentrations over 48 h. Applying an unsupervised multivariate analysis, no clear separation of control and high concentration samples was observed, with the PCA score plot dominated by temporal changes in the (baseline) metabolome (Fig. 3a). Samples from both the highest concentration exposure group and the controls followed the same pattern as observed in Fig. 2a, with temporal separation along PC2. However, the effects of chemical exposure were evident when the nESI-DIMS data were analysed using univariate statistics (one-way ANOVA, Fig. 3b). While minimal (ca. only 20–30) features changed significantly at 1 and 2 h (adjusted p-values ≤ 0.05), a large metabolic perturbation was discovered at 6 h (almost 900 features changing significantly, or 20% of the detected metabolome), an effect which decreased somewhat at later time points. Considering the results in Fig. 2b and 3b, it is possible that early metabolic changes induced by CdCl_2_ are being masked by the large changes in the baseline metabolome following the media change, up to 6 h into the exposure study. The dataset from this study was putatively annotated based on accurate mass using an in-house HMDB-based list of metabolites revealing 418 putative annotations of 4983 spectral feature in the dataset (these annotations are included in the Supplementary Information). The changes in the intensity of putatively annotated features of glutathione were further investigated across time and concentration of CdCl_2_. These features corresponded to two adducts [M-H]^−^ and [M + K-2H]^−^ (Fig. SI-7 and Fig. SI-8). The change in the glutathione peak intensity was predominantly associated with the sampling time, with the control and toxicant-exposed samples behaving relatively similarly. While changes in the peak intensity due to CdCl_2_ exposure at each time point were more subtle, the medium exposure concentration did induce a significant decrease for the feature corresponding to [M-H]^−^ of glutathione at 6 h, relative to control samples (adjusted p-value ≤ 0.05).

To conclude, these observations confirmed that the HTS metabolomics workflow can detect toxicant-induced changes in the metabolome, while also providing insights into the optimal sampling times of the HepaRG, albeit for a single model toxicant. Specifically, the variability in the baseline metabolome may confound the ability to discover the effects of toxicant exposure after 1 and 2 h due to the metabolic changes induced by the media change.

### Modification of solvent system to extract polar metabolites and lipids

An HTS metabolomics workflow requires every step (including sample extraction) to be relatively rapid, for example through parallelisation and/or automation. While extraction of both polar metabolites and lipids often uses biphasic solvent systems, monophasic extractions are more straightforward to automate and therefore more appropriate for HTS (Southam et al., 2017; Wu et al., 2008). In addition, biphasic extractions can partition metabolites across both solvent phases and increase the risk of metabolite loss within the interphase layer, both of which are particularly problematic for low biomass samples (Kapoore & Vaidyanathan, 2016). Here, a monophasic solvent system consisting of 1:3:1 (v/v/v) water:methanol:chloroform was used (above), which was a modification of the classic monophasic solvent system 0.8:2:1 (v/v/v) water:methanol:chloroform (Bligh & Dyer, 1959) because a separation of polar and non-polar solvent phases was occasionally observed for this classic method (likely due to intracellular water, salts or residual wash solvent). The ratio of 1:3:1 (v/v/v) water:methanol:chloroform has been reported previously in metabolomics studies (Baptista et al., 2018; van der Hooft et al., 2019) and, as shown above, was effective at extracting polar metabolites for positive and negative ionisation modes by nESI-DIMS. However, attempts to analyse lipids in these extracts resulted in unacceptable electrospray instability (data not shown) that was likely due to the presence of more polar metabolites, prompting the evaluation of separate monophasic extractions of polar metabolites and lipids in a manner that would allow for automation.

**Fig. 4:**
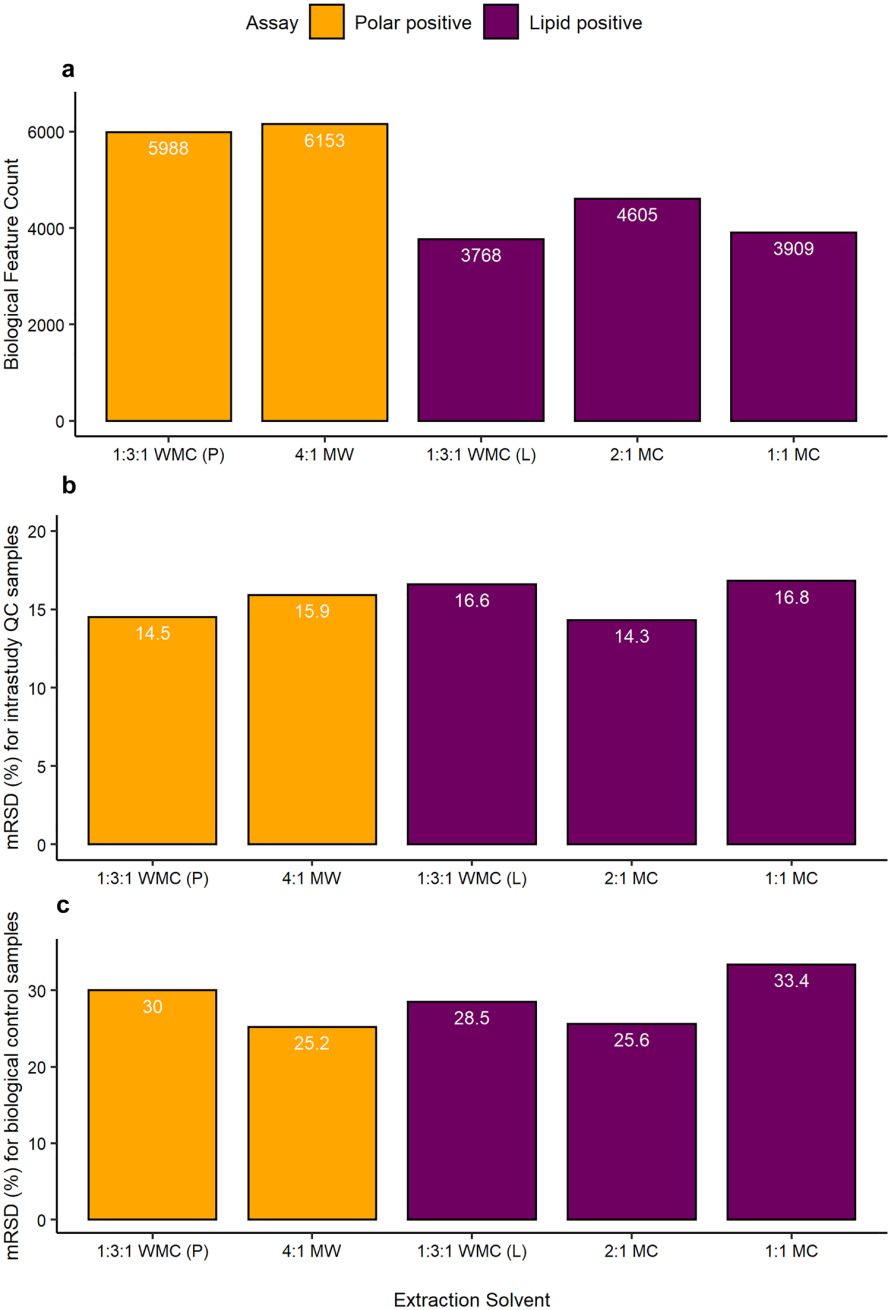
Assessment of **a** relative sensitivity, **b** analytical repeatability of intrastudy QCs, and **c** total repeatability of biological samples of the in vitro HTS nESI-DIMS (positive ionisation mode) metabolomics workflow, comparing different extraction solvent systems of the 96-well microplates with 50,000 HepaRG per well. For polar metabolites (yellow bars), 1:3:1 (v/v/v) water:methanol:chloroform and 4:1 (v/v) methanol:water were compared, while for lipids (purple bars), 1:3:1 (v/v/v) water:methanol:chloroform, 2:1 (v/v) methanol:chloroform and 1:1 (v/v) methanol:chloroform were investigated

To extract polar metabolites, two monophasic solvent systems were compared: 1:3:1 (v/v/v) water:methanol:chloroform and 4:1 (v/v) methanol:water, the latter being a well-established method and using the same solvent ratio as for the resuspension of dried extracts prior to nESI-DIMS. Again, feature count and mRSD values of biological and intrastudy QCs were used to assess data quality. Of the two solvent systems, 4:1 methanol:water (v/v) yielded slightly higher feature count after blank subtraction and lower mRSD in biological samples, hence it was selected as the more optimal for future use (Fig. 4). Possible causes of the elevated variation associated with the chloroform-containing extracts include partial evaporation of the already low volumes of this volatile solvent, and/or co-extraction of more lipophilic species that interfered with nanoelectrospray ionisation. To extract lipids, three monophasic solvent systems were compared: 1:3:1 (v/v/v) water:methanol:chloroform, 2:1 (v/v) methanol:chloroform and 1:1 (v/v) methanol:chloroform. The latter two were proposed to increase the percentage of chloroform, thus maximising lipid extraction. In addition, 2:1 (v/v) methanol:chloroform is used to resuspend dried extracts prior to nESI-DIMS and should minimise any potential problems associated with resolubilisation. The 2:1 (v/v) methanol:chloroform yielded ca. 20% higher feature count than the other solvent systems, and the lowest mRSD values for both intrastudy QCs and biological samples (Fig. 4). These results met the workflow criteria for sensitivity and variability, and thus the 2:1 (v/v) methanol:chloroform solvent system was selected for extraction of lipids. The datasets corresponding to the optimal extraction solvents were putatively annotated using accurate mass revealing 366 putative annotations out of 6153 spectral features for polar metabolites extracted with 4:1 methanol:water (v/v), whilst for lipids extracted with 2:1 methanol:chloroform (v/v), 892 out of 4605 spectral features were putatively annotated (these annotations are included in the SI – Table SI-3 and Table SI-4).

## Conclusions

A newly established, semi-automated in vitro metabolomics workflow for the extraction and analysis of intracellular metabolites from hepatocytes of HepaRG has demonstrated compatibility with a high-throughput screening platform, based on only 50,000 cells per well in a 96-well format. Although the initially proposed monophasic solvent system (1:3:1 (v/v/v) water:methanol:chloroform) achieved the target criteria for sensitivity and repeatability when used for polar nESI-DIMS assays (both positive and negative ion modes), issues with spray stability during lipid analysis were encountered prompting an evaluation of separate extraction methods for polar metabolites and lipids that achieved the target criteria when using 4:1 (v/v) methanol:water for polar metabolites and 2:1 (v/v) methanol:chloroform, for lipids. Focusing on polar intracellular metabolites (1:3:1 (v/v/v) water:methanol:chloroform solvent system), the analytical and informatics workflows were demonstrated in a toxicological study with CdCl_2_. First, the effect of time on the baseline metabolism of unexposed hepatocytes of HepaRG was characterised, highlighting the metabolic variability during the initial 6 h following a media change. In addition, this study confirmed that the nESI-DIMS approach is sufficiently sensitive to detect toxicant-induced changes in the intracellular metabolome, using only 50,000 HepaRG per sample. While developed for applications in toxicology, this in vitro HTS nESI-DIMS metabolomics workflow is applicable to a wide range of screening applications that demand high sample throughput with limited numbers of cells.

## Supplementary Information

Below is the link to the electronic supplementary material.

Supplementary file1 (DOCX 409 kb)

Supplementary file2 (XLSX 246 kb)

## Data Availability

The datasets from this study are available from the corresponding author on request.
