# Supplementary material for: Integrating in vitro metabolomics with a 96-well high-throughput screening platform
Source: Metabolomics. 2022 Jan 9;18(1):11. doi: 10.1007/s11306-021-01867-3 (PMC8743266; doi:10.1007/s11306-021-01867-3)
Supplement: Supplementary file 1 — Supplementary file1 (DOCX 409 kb) [file 11306_2021_1867_MOESM1_ESM.docx]

SUPPLEMENTARY MATERIAL FOR:

**Integrating *in vitro* metabolomics with a 96-well high-throughput screening platform**

**Julia M. Malinowska^1^, Taina Palosaari^2^, Jukka Sund^2^, Donatella Carpi^2^, Mounir Bouhifd^2,*^, Ralf J.M. Weber^1,3^, Maurice Whelan^2^ and Mark R. Viant ^1,3,Ϯ^**

^1^ School of Biosciences, University of Birmingham, Birmingham, B15 2TT, UK

^2^ European Commission, Joint Research Centre (JRC), 21027 Ispra, Italy

^3^ Phenome Centre Birmingham, University of Birmingham, Birmingham, B15 2TT, UK

**^Ϯ^** Correspondence: [m.viant@bham.ac.uk](mailto:m.viant@bham.ac.uk); Tel.: +44 (0)121 414 2219

**Table of Contents**

[Supplementary Methods 3](#_Toc89356780)

[Fig. SI-1 Layout of 96-well microplates employed for evaluation of the in vitro metabolomics-based workflow and modifications to the solvent system. 3](#_Toc89356782)

[Fig. SI-2 Layout of 96-well microplates employed for a proof-of-principle toxicological study using the in vitro metabolomics-based workflow 4](#_Toc89356783)

[Cell culturing and exposure to CdCl_2_ 5](#_Toc89356784)

[Assessments of sensitivity and intra- and inter-plate metabolic variability: Processing and analysis of DIMS metabolomics data 5](#_Toc89356785)

[Pilot study into effects of CdCl_2_ on the HepaRG metabolome: Processing and analysis of DIMS metabolomics data 5](#_Toc89356786)

[Modification of solvent system to extract polar metabolites and lipids 6](#_Toc89356787)

[Supplementary Results 7](#_Toc89356788)

[Fig. SI-3 PCA plots of control samples across 5 time points showing the separation of samples occurs alongside PC2 (i.e. a separation of earlier and later time points). 7](#_Toc89356789)

[Fig. SI-4 Temporal changes in the metabolome of unexposed HepaRG up to 48 h after a media change 8](#_Toc89356790)

[Fig. SI-5 Venn diagram of statistically significant metabolic features between each pair of consecutive time points over 48 h, for unexposed HepaRG 9](#_Toc89356791)

[Fig. SI-6 Changes in the intensity of putatively annotated peak of glutathione (([M+K-2H]- adduct),^12^C isotope) over time, in unexposed (control) HepaRG samples 10](#_Toc89356793)

[Fig. SI-7 Changes in the intensity of the putatively annotated peak of glutathione (([M-H]- adduct),^12^C isotope) over 48 h and across three concentrations of cadmium chloride as well as control (unexposed) hepatocytes of HepaRG 11](#_Toc89356794)

[Fig. SI-8 Changes in the intensity of the putatively annotated peak of glutathione (([M+K-2H]- adduct),^12^C isotope) over 48 h and across three concentrations of cadmium chloride as well as control (unexposed) hepatocytes of HepaRG 12](#_Toc89356795)

[References 13](#_Toc89356796)

**Supplementary Methods**

##
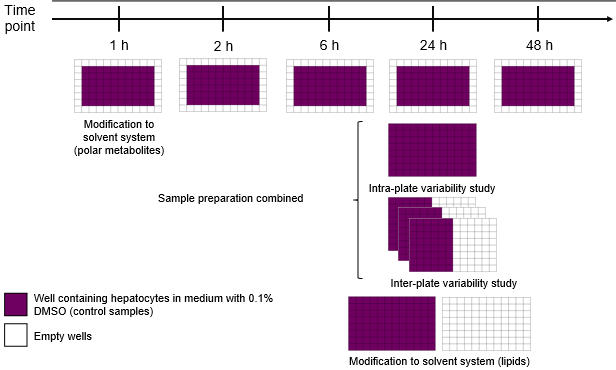


## **Fig. SI-1** Layout of 96-well microplates employed for evaluation of the in vitro metabolomics-based workflow and modifications to the solvent system. The assessment of sensitivity and intra-plate metabolic variability was conducted using hepatocytes in all 96 wells incubated for 24 h, while the assessment of sensitivity and inter-plate metabolic variability employed hepatocytes in 48 wells per 96-well microplate, also incubated for 24 h. Modifications to the solvent system used to extract polar metabolites were performed using hepatocytes in 60 wells per 96-well microplate, incubated for 1 h


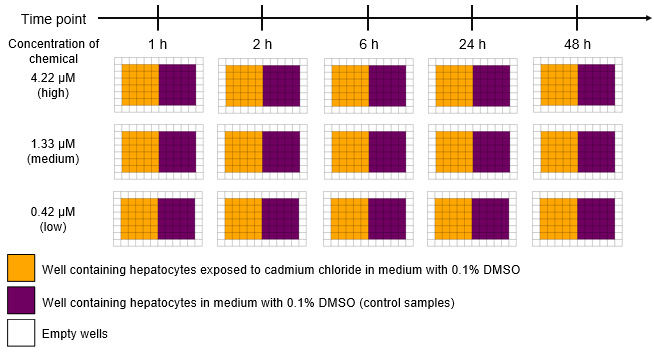


## **Fig. SI-2** Layout of 96-well microplates employed for a proof-of-principle toxicological study using the in vitro metabolomics-based workflow. Three concentrations of the model toxicant (cadmium chloride) were employed across five time points. Control samples (unexposed hepatocytes of HepaRG) were present in every microplate

## ***Cell culturing and exposure to CdCl_2_***

The experiments employed HepaRG, human hepatic cell line, established by the INSERM laboratory (National Institute of Health and Medical Research) in Rennes, France. For this study, Biopredic International (Rennes, France) provided undifferentiated HPR101 HepaRG cells in cryopreserved vials (batch HPR-101056). Culturing of undifferentiated HepaRG in polystyrene 96-well microplates was conducted as previously described by Joossens et al. (2019) using 5 x 10^4^ cells/well in 100 μL medium, except that cells were incubated in DMSO-free medium for 72 h prior to treatment. Wells without cells served as extraction blanks. Sample configurations in the microplates differed depending on the study (see Fig. SI-1, SI-2 and the results section in the main paper). On the day of experimentation, DMSO-free medium was changed to medium containing 0.1% DMSO (v/v (Sigma, Milan, Italy)) - for control samples - or medium containing cadmium chloride (Sigma) at 0.42 μM, 1.33 μM, and 4.22 μM referred to here as ‘low’, ‘medium’ and ‘high’ concentrations. Cells were incubated for 1, 2, 6, 24 and 48h (n=6 replicates per time point). The viability of the HepaRG cell line was not affected by cadmium chloride when exposed to the highest concentration of this chemical at 4.22 µM at the time points tested in this study. At each time point, the medium was removed and discarded, and the adherent hepatocytes were washed twice with 180 μL of 0.9% ice-cold sodium chloride (w/v (Fresenius Kabi, Isola della Scala, Italy)) and once with 200 μL of ice-cold sterile-filtered water (Sigma). All washes occurred in under 20 s using ELx405 microplate washers (BioTek Instruments, Winooski, VT, USA), with the last wash to remove salts prior to DIMS analysis (Deng et al., 2018). Microplates were sealed using adhesive foil (Sigma; for polar metabolite extractions) or heat-sealed foil (180 °C for 5 s; Bio-Rad, Milan, Italy; for lipid extractions), frozen on dry ice (the metabolism was quenched when placed on dry ice (after cell media removal and washing), and stored at - 80 °C.

## ***Assessments of sensitivity and intra- and inter-plate metabolic variability: Processing and analysis of DIMS metabolomics data***

DIMS data were processed using the DIMSpy tools within the Galaxy workflow management system (Southam et al., 2017; Weber & Zhou, 2020). Parameters employed for the data processing were as follows: signal-to-noise ratio and minimum number of scans required for each *m*/*z* window or event were set to 3. A minimum fraction of scans a feature had to be present in was 0.75, using a 2 ppm error tolerance for *m/z* deviation. The DIMSpy ‘replicate filter’ tool was skipped due to the new internal scan replication method. The samples were aligned, using the align samples tool, by applying a ppm error tolerance of 2 and 3, for polar positive and negative assays, respectively. Only features which passed blank filtering (the ratio between non-blank and blank peak intensities set to 10) in at least 80% of non-blank samples were retained. The dataset was then filtered to keep only the features present in 80% of study samples including both biological and intrastudy QC samples. A missing value sample filter was then applied using a 20% threshold, and samples not meeting this threshold (i.e., sample has >20% missing values) were removed. After processing, probabilistic quotient normalisation (PQN) was applied and the sensitivity and reproducibility of the data were evaluated (Dieterle et al., 2006).

## ***Pilot study into effects of CdCl_2_ on the HepaRG metabolome: Processing and analysis of DIMS metabolomics data***

DIMS data were processed using the DIMSpy tools within the Galaxy workflow management system from the peak picking step (Southam et al., 2017; Weber & Zhou, 2020), as described above, with the exception of the removal of peaks from the extraction blanks; here the ratio between non-blank and blank peak intensities was set to 15 for the polar assay in negative ionisation mode. The step was followed by retaining features present in 80% of study samples as before. The next steps were conducted using the R package structToolbox (Lloyd & Weber, 2021). Samples containing greater than 20% of missing values were removed, while only features present in at least 80% of QC samples were retained. The resulting peak intensity matrix was corrected for signal drift, and normalised using the PQN method (Dieterle et al., 2006). Features which RSDs exceeded 30% in QC samples were removed. This matrix was then used for univariate analysis as described below. For principal component analysis (PCA), the missing values were imputed using k-nearest neighbour algorithm (k=5) following generalised log transformation and mean centring. PCA identified five outliers that were removed and data was reprocessed without the outlying samples.

For the data analysis, each exposure group had 6 replicates, whilst the control group had 18 replicates (due to the control samples being on three microplates per time point as shown in Fig. SI-2). The univariate analysis employed was Analysis of Variance (ANOVA) with a p-value threshold set to 0.05 and false discovery rate correction using the Benjamini-Hochberg procedure. The step was followed by post-hoc testing using the Tukey-Kramer method as used for unbalanced designs with a p-value also set to 0.05. It was pre-defined that at least four measurements had to be present per feature in a tested group if possible (one group, low concentration of chemical at 24 h had only 3 replicates retained so that was reflected for all groups at this time point). In the post-hoc testing, the multiple test correction method was based upon filtering features with ANOVA (i.e. the features with FDR-corrected p-values ≤ 0.05 from ANOVA were used to investigate the outcome of post-hoc testing).

## ***Modification of solvent system to extract polar metabolites and lipids***

For polar metabolites, two solvent systems were compared: 1:3:1 (v/v/v) water:methanol:chloroform and 4:1 (v/v) methanol:water. The former was conducted as described earlier except for adding a pre-made 75% methanol:water instead of adding them separately. The latter employed 60 μL of 80% methanol for both the extraction and re-extraction steps with 40 μL and 60 μL of extract removed and pooled, per well. All other steps, including bioshaking, centrifuging and drying, were conducted as described in the main paper. For lipids, three monophasic solvent systems were compared: 1:3:1 (v/v/v) water:methanol:chloroform, 2:1 (v/v) methanol:chloroform and 1:1 (v/v) methanol chloroform. The former was conducted as described above. For 2:1 and 1:1 (v/v) methanol:chloroform, 60 μL of methanol was added to each well, the extract (40 μL) was transferred to a clean polypropylene microplate, the original well re-extracted (40 μL methanol), and this extract (40 μL) was transferred to the same well of the polypropylene 96-well microplate (“collection plate”). Chloroform was then added: 40 and 80 μL for 2:1 (v/v) and 1:1 (v/v) methanol:chloroform, respectively. The subsequent steps were as described before. The volumes taken for drying corresponded to 80% of the extract volume in the final polypropylene plate: 80 μL, 96 μL and 128 μL corresponding to 1:3:1 (v/v/v) water:methanol:chloroform, 2:1 (v/v) methanol:chloroform and 1:1 (v/v) methanol:chloroform, respectively. For polar DIMS assays, individual wells were resuspended and analysed as described above. For lipidomics, extracts were resuspended in 40 μL of 2:1 (v/v) 7.5 mM methanolic ammonium acetate:chloroform (final concentration of 5 mM ammonium acetate), centrifuged, and 20 μL were taken for analysis. Intrastudy QCs were generated after sample resuspension by pooling representative wells, then re-aliquotting for DIMS analysis. For DIMS analyses of lipids (positive ionisation mode), each sample was analysed as a single infusion using internal scan replication with an infusion volume of 12 μL. The acquisition parameters were as reported previously (Southam et al., 2017) except the nESI voltage was +1.3 kV, and volume of air to aspirate after sample was 2.5 μL. The principal data processing parameters and analyses were as used above.

# **Supplementary Results**


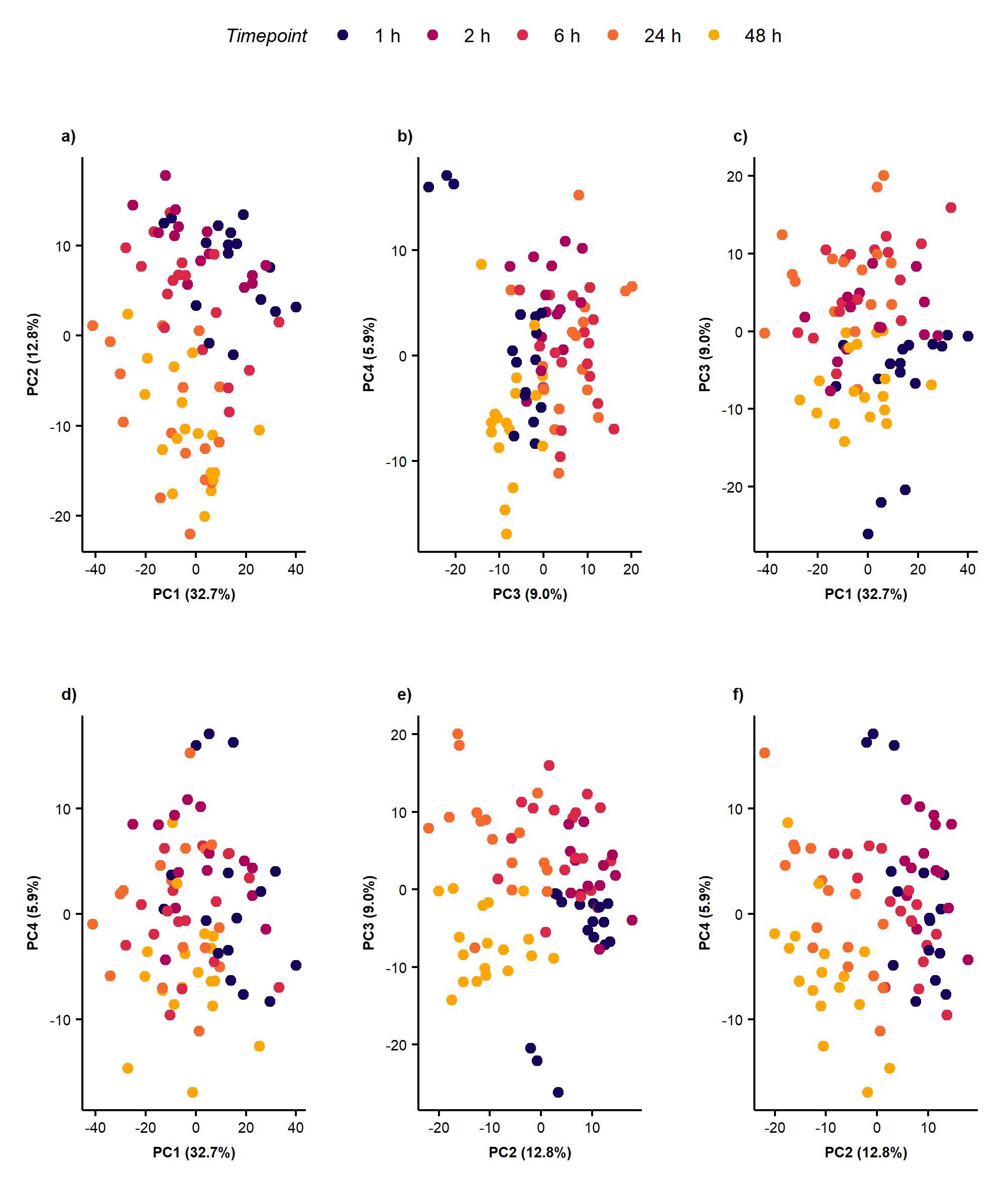


## **Fig. SI-3** PCA plots of control samples across 5 time points showing the separation of samples occurs alongside PC2 (i.e. a separation of earlier and later time points).

**
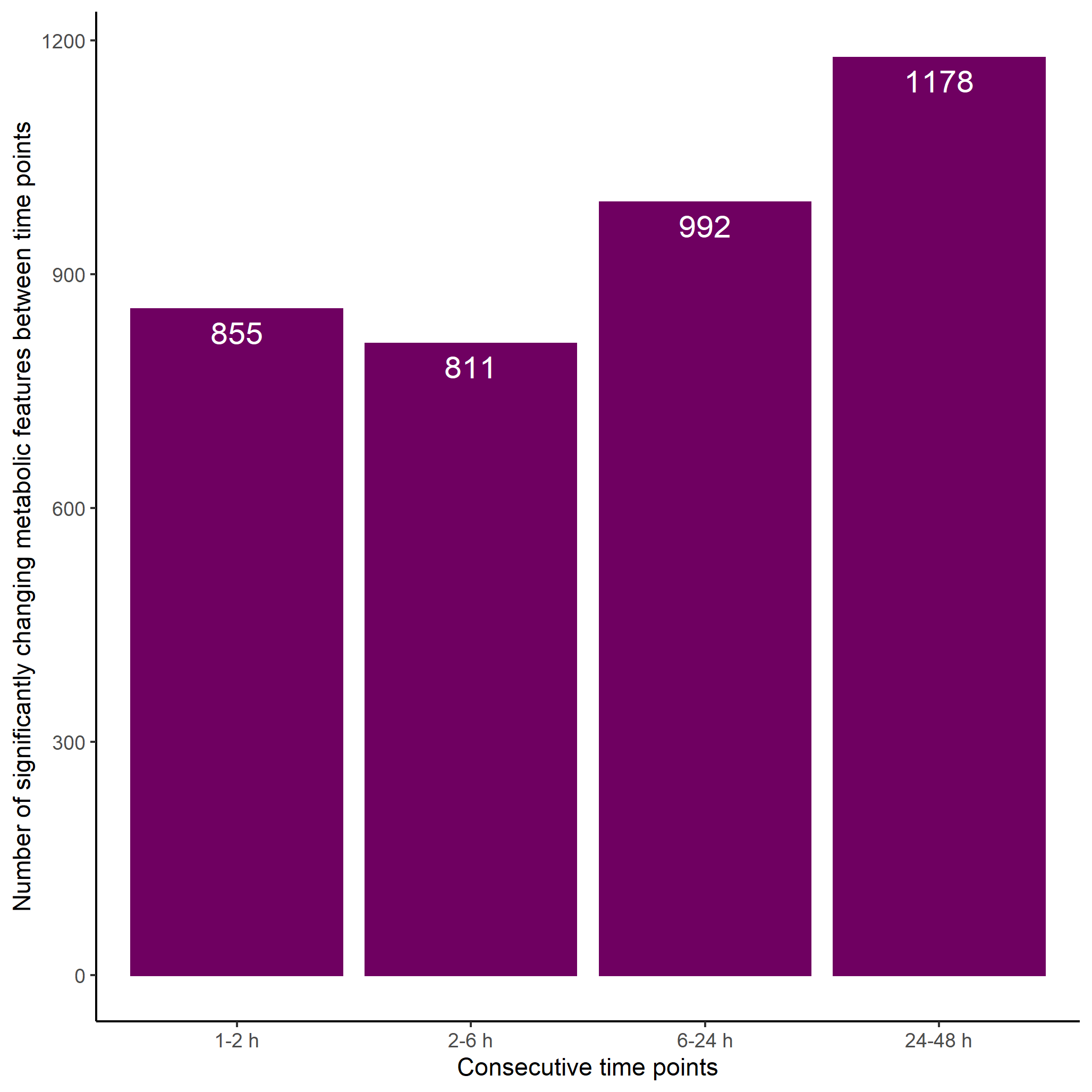
**

## **Fig. SI-4** Temporal changes in the metabolome of unexposed HepaRG up to 48 h after a media change: Number of significantly changing metabolic features between each pair of consecutive time points (one-way ANOVA, FDR-corrected p ≤0.05 with the Tukey-Kramer method for post-hoc testing as used for unbalanced designs)


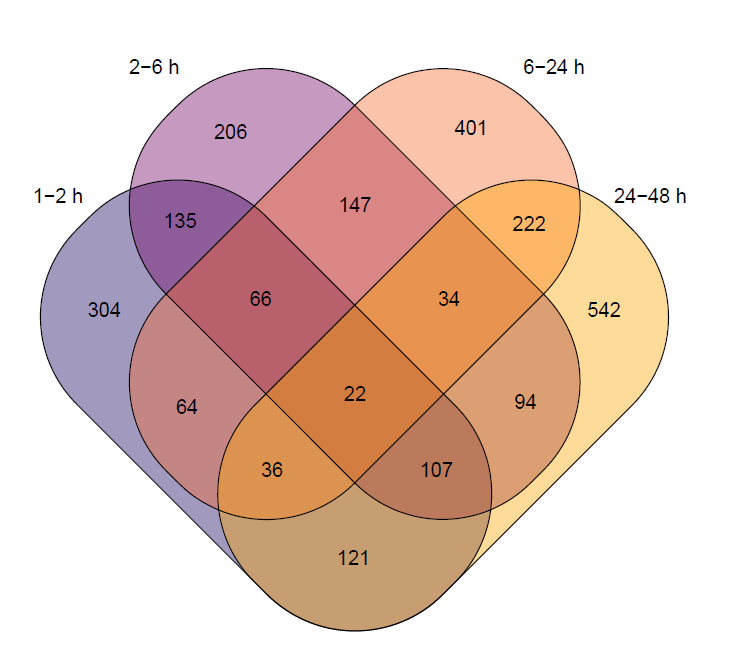


## **Fig. SI-5** Venn diagram of statistically significant metabolic features between each pair of consecutive time points over 48 h, for unexposed HepaRG (one-way ANOVA, FDR-corrected p ≤0.05 with the Tukey-Kramer method for post-hoc testing as used for unbalanced designs)

## **
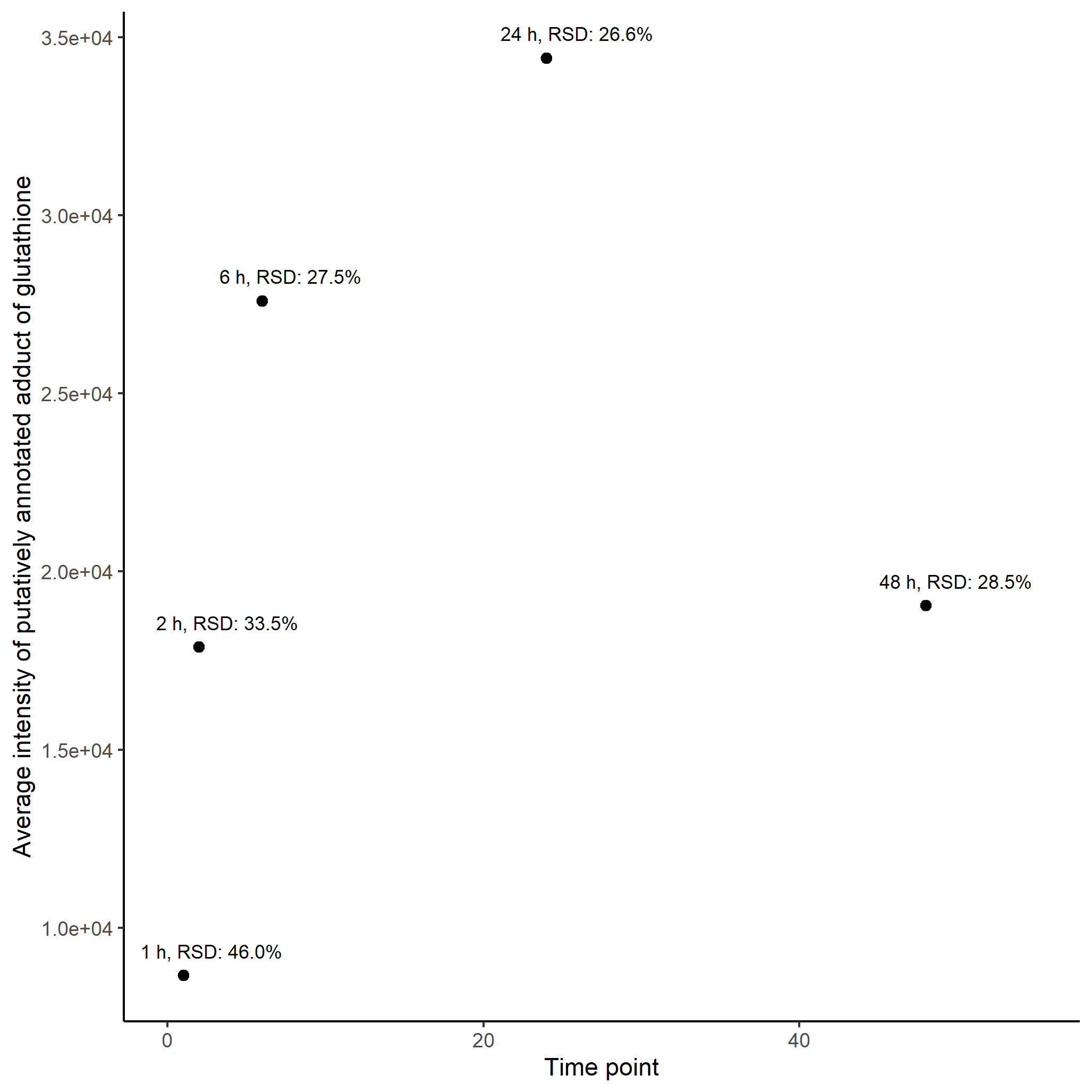
**

## **Fig. SI-6** Changes in the intensity of putatively annotated peak of glutathione (([M+K-2H]^-^ adduct),^12^C isotope) over time, in unexposed (control) HepaRG samples. RSDs (%) of the peak intensities, per time point group, are shown next to the data point


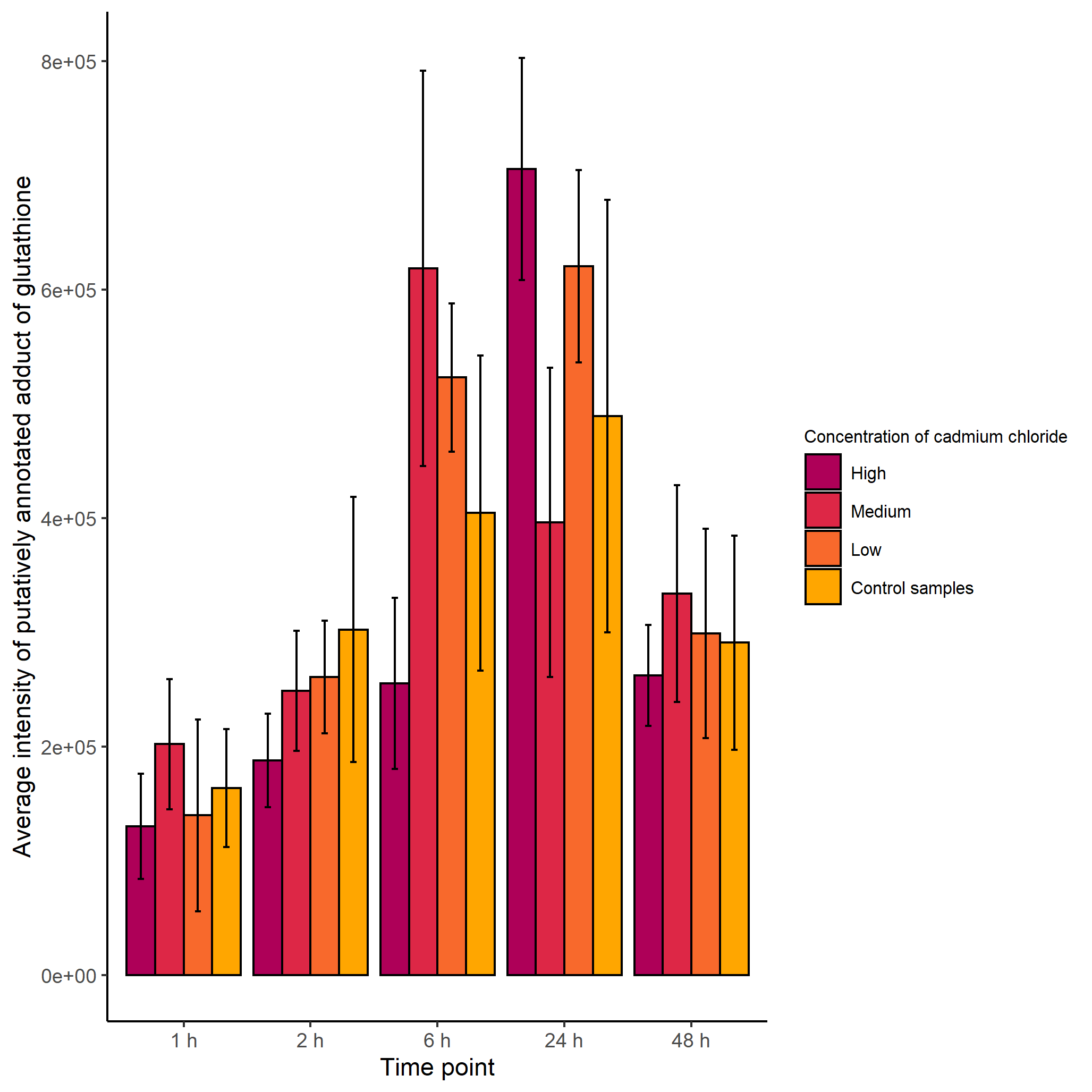


## **Fig. SI-7** Changes in the average intensity of the putatively annotated peak of glutathione (([M-H]^-^ adduct),^12^C isotope) over 48 h and across three concentrations of cadmium chloride as well as control (unexposed) hepatocytes of HepaRG. Error bars represent one standard deviation

##
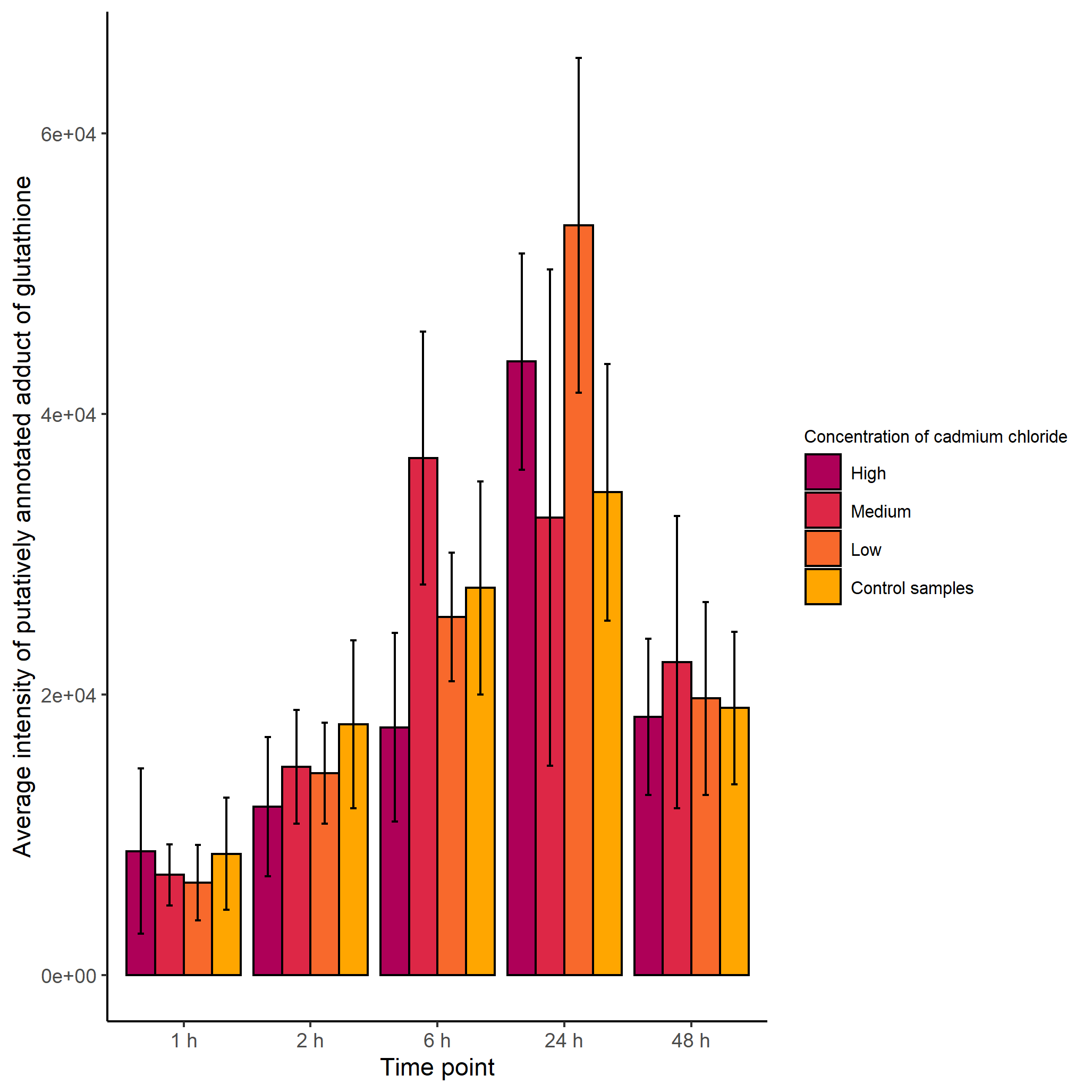
**Fig. SI-8** Changes in the average intensity of the putatively annotated peak of glutathione (([M+K-2H]^-^ adduct),^12^C isotope) over 48 h and across three concentrations of cadmium chloride as well as control (unexposed) hepatocytes of HepaRG. Error bars represent one standard deviation

# **References**

Deng, J., Zhang, G., & Neubert, T. A. (2018). Metabolomic Analysis of Glioma Cells Using Nanoflow Liquid Chromatography–Tandem Mass Spectrometry. *Methods in Molecular Biology*, *1741*, 125–134.

Dieterle, F., Ross, A., Schlotterbeck, G., & Senn, H. (2006). Probabilistic quotient normalization as robust method to account for dilution of complex biological mixtures. Application in1H NMR metabonomics. *Analytical Chemistry*, *78*(13), 4281–4290. https://doi.org/10.1021/ac051632c

Joossens, E., Macko, P., Palosaari, T., Gerloff, K., Ojea-Jiménez, I., Gilliland, D., Novak, J., Fortaner Torrent, S., Gineste, J. M., Römer, I., Briffa, S. M., Valsami-Jones, E., Lynch, I., & Whelan, M. (2019). A high throughput imaging database of toxicological effects of nanomaterials tested on HepaRG cells. *Scientific Data*, *6*(1), 1–10. https://doi.org/10.1038/s41597-019-0053-2

Lloyd, G. R., & Weber, R. J. M. (2021). *structToolbox: Data processing & analysis tools for Metabolomics and other omics. R package version 1.4.2.*

Southam, A. D., Weber, R. J. M., Engel, J., Jones, M. R., & Viant, M. R. (2017). A complete workflow for high-resolution spectral-stitching nanoelectrospray direct-infusion mass-spectrometry-based metabolomics and lipidomics. *Nature Protocols*, *12*(2), 310–328. https://doi.org/10.1038/nprot.2016.156

Weber, R. J., & Zhou, J. (2020). DIMSpy: Python package for processing direct-infusion mass spectrometry-based metabolomics and lipidomics data (Version v2.0.0). *Zenodo*. https://doi.org/http://doi.org/10.5281/zenodo.3764169
